# Genome-Wide Association Study of Meat Quality Traits in a Three-Way Crossbred Commercial Pig Population

**DOI:** 10.3389/fgene.2021.614087

**Published:** 2021-03-17

**Authors:** Guangxiong Gao, Ning Gao, Sicheng Li, Weijian Kuang, Lin Zhu, Wei Jiang, Weiwei Yu, Jinbiao Guo, Zhili Li, Chengzhong Yang, Yunxiang Zhao

**Affiliations:** ^1^School of Life Sciences and Engineering, Foshan University, Foshan, China; ^2^State Key Laboratory of Biocontrol, School of Life Sciences, Sun Yat-sen University, Guangzhou, China; ^3^Guangxi Yangxiang Co., Ltd., Guigang, China

**Keywords:** genome-wide association study, crossbred pigs, meat quality, moisture, conductivity, marbling score, meat color, intramuscular fat content

## Abstract

Meat quality is an important trait for pig-breeding programs aiming to meet consumers’ demands. Geneticists must improve meat quality based on their understanding of the underlying genetic mechanisms. Previous studies showed that most meat-quality indicators were low-to-moderate heritability traits; therefore, improving meat quality using conventional techniques remains a challenge. Here, we performed a genome-wide association study of meat-quality traits using the GeneSeek Porcine SNP50K BeadChip in 582 crossbred Duroc × (Landrace × Yorkshire) commercial pigs (249 males and 333 females). Meat conductivity, marbling score, moisture, meat color, pH, and intramuscular fat (IMF) content were investigated. The genome-wide association study was performed using both fixed and random model Circulating Probability Unification (FarmCPU) and a mixed linear model (MLM) with the rMVP software. The genomic heritability of the studied traits ranged from 0.13 ± 0.07 to 0.55 ± 0.08 for conductivity and meat color, respectively. Thirty-two single-nucleotide polymorphisms (SNPs) were identified for meat quality in the crossbred pigs using both FarmCPU and MLM. Among the detected SNPs, five, nine, seven, four, six, and five were significantly associated with conductivity, IMF, marbling score, meat color, moisture, and pH, respectively. Several candidate genes for meat quality were identified in the detected genomic regions. These findings will contribute to the ongoing improvement of meat quality, meeting consumer demands and improving the economic outlook for the swine industry.

## Introduction

Meat quality, a comprehensive indicator that includes moisture, intramuscular fat (IMF), pH, meat color, water-holding capacity, marbling, and tenderness (Noidad et al., 2019), is among the most important traits in the swine industry. In addition to genetics, non-genetic factors, such as species, management, and environmental background, affect meat quality (Womack et al., 2012). Historically, swine research efforts focused on growth performance but neglected meat quality. However, as living standards improve globally, more consumers are prioritizing meat quality. Consequently, pig farmers are interested in improving meat quality to meet the new meat-market demands (Nonneman et al., 2013).

Multiple genes, including major genes and genes with moderate or minor effects, control meat quality. *RN*, *RKAG3*, *RYR1*, *PHKG1*, *MC4R*, and *insulin-like growth factor 2 (IGF2)* are the major genes reported to affect meat-quality traits (Milan et al., 2000; Barbut et al., 2008; Yu et al., 2008; Oczkowicz et al., 2013; Ma et al., 2014; Lu et al., 2018). In total, 30,580 quantitative trait loci (QTLs) were released for public access on the pig QTL database^1^, which reported 691 pig traits associated with meat quality. Previous research identified many candidate genes for meat-quality traits, including *adenylosuccinate lyase* (*ADSL*) associated with drip loss and pH (Ramos et al., 2006; Karol et al., 2010) and *ubiquitin-specific peptidase 43* (*USP43*) associated with five meat-quality traits, including IMF, marbling, moisture, meat color, and color score (Luo et al., 2012). Some regions were identified for multiple traits, such as on SSC6 from 28 to 29.5 Mb for purge and IMF containing the candidate genes glucose-6-phosphate isomerase (GPI) and KCTD15 (Nonneman et al., 2013). The BDKRB2 and UTRN genes were identified to associate with IMF in Duroc population using single-locus and multi-locus genome-wide association studies (GWASs) (Ding et al., 2019). The MYCT1 and BINP3 genes were found to associate with both meat color and pH in Qingyu pigs (Wu et al., 2020). Additionally, most QTLs have been identified using linkage mapping, thus representing large chromosomal regions (Varona et al., 2002). As high-density single-nucleotide polymorphism (SNP) arrays become more accessible, GWASs are being widely used to identify candidate genes despite most meat-quality traits exhibiting low-to-moderate heritability (Hermesch et al., 2000; Suzuki et al., 2005). Further exploration of meat-quality-related genes remains necessary owing to the insufficient research on gene localization of meat-quality traits.

Many breeding enterprises favor crossbred Duroc × (Landrace × Yorkshire) pigs [D (LY)] for their high feed-utilization rates and large eye muscle area, while meat quality is often neglected. Meat-quality traits are difficult to measure and cannot be assessed without slaughter, which greatly increases the difficulty and cost of breeding programs selecting for meat quality. In the present study, a GWAS was conducted using the Porcine SNP50 Genotyping BeadChip to identify QTLs for meat-quality traits in a crossbred D (LY) porcine population. This study was conducted to identify candidate genes and potential breeding markers and more deeply investigate the genetic architecture of meat-quality traits.

## Materials and Methods

### Ethics Statement

All experimental animals were handled in accordance with the guidelines of the Institutional Animal Care and Use Committee of Foshan University. The Institutional Animal Care and Use Committee of Foshan University approved this study.

### Animals

We collected 582 D (LY) commercial pigs (249 males and 333 females) from two farms (Fengda and Xinglin) of Guangxi Yangxiang Co., Ltd. These pigs were offspring of 45 boars and 96 sows. The pigs were reared under the same management conditions and similar environments, with automatic water and free food intake (with the nutritional formula shown in Table 1). Boars and sows were raised separately, and the young boars were castrated 6–7 days after birth. The pigs were slaughtered in the same commercial abattoir at 150 ± 3 days of age.

**TABLE 1:** Nutritional formula of the D (LY) population.

**Components**	**Content**	
	**30∼70 kg**	**70 kg∼Live weight**
Energy, MJ/kg	3,292	3,291
Moisture,%	11.74	11.61
Crude protein,%	15.5	15.0
Crude fat,%	1.83	1.68
Calcium,%	0.60	0.55
SID Lys,%	0.90	0.77
SID Met,%	0.27	0.18
SID Trp,%	0.12	0.10

### Phenotypes

Trained personnel recorded the phenotypic data for six meat-quality traits per individual pig as per the guidelines of the National Pork Producers Council (NPPC, 1991) of the United States. All meat-quality measurements were taken on the left side of the carcass. Meat color was measured as follows: (1) grayish white (abnormal flesh color), (2) mild gray (inclined to abnormal flesh color), (3) normal bright red, (4) slightly dark red (normal flesh color), and (5) dark purple (abnormal flesh color). Marbling score was assessed from 1 to 5. Both measurements were assessed subjectively via the longissimus muscle (LM) according to the NPPC. pH was measured via the LM using a Delta 320 pH meter (Mettler Toledo, Columbus, OH, United States) 45 min after slaughter. IMF was determined from the thoracic lumbar LM via Soxhlet petroleum-ether extraction. Moisture was analyzed via the thoracic lumbar LM by routine oven drying. Conductivity was measured via the dorsal LM between the 13th and 14th ribs using the LF-STAR conductivity meter (Matthaus, Pottmes, Germany). Meat color, pH, marbling score, and conductivity were measured in triplicate for each sample, and the average of the three measurements was used.

### Genotyping and Quality Control

DNA was extracted from the ear tissue using a genome extraction kit (Wuhan NanoMagBio Technology Co., Ltd., China). DNA quality was assessed by measuring the light absorption ratios (A260/280 and A260/230) at ≥40 ng/μl. Genomic DNA was genotyped on the GeneSeek Porcine 50K SNP Beadchip (GeneSeek, Lansing, MI, United States). Quality control of the SNP data was conducted using PLINK software (Purcell et al., 2007). Briefly, individuals with call rates >0.95 and markers with call rates >0.99, minor allele frequencies (MAF) >0.05, and Hardy–Weinberg (HWE) *P* > 10^–4^ were retained. All markers located on sex chromosomes or in unmapped regions were excluded. Missing genotypes were imputed using the Beagle software (Browning and Browning, 2009). After quality control, 34,057 SNPs were used for subsequent analyses. Supplementary Table 1 shows the SNP distribution after data quality control and the average distance between adjacent SNPs on each chromosome.

### Statistical Analyses

Genomic heritability of the meat-quality traits was calculated by dividing the genetic variance by the sum of the genetic and residual variances using the hiblup package (Yin et al., 2019). The model can be written as follows:

y=Xb+Zu+e

where *y* is the vector of phenotypic values; *b* is a vector of fixed effects, including sex, farm of origin, and batch containing the year-season effect; and *u* represents breeding values. *X* and *Z* were design matrices for *b* and *u*, respectively; *e* represents the residual error vector. In this study, $u∼N\left(0,G\sigma_{u}^{2}\right)$, in which $\sigma_{u}^{2}$ is the unknown additive genetic variance, and *G* is the genomic relationship matrix (VanRaden, 2008).

Association analysis was performed using the fixed and random model Circulating Probability Unification (FarmCPU) (Liu et al., 2016) and mixed linear model (MLM) (Price et al., 2006) with the rMVP software (Yin et al., 2020). The FarmCPU model iteratively uses the fixed and random effects to simultaneously control false positives and false negatives. The model can be written as follows:

$$y=Tw_{i}+P_{j}q_{j}+m_{k}h_{k}+e,$$

where *y* is the vector of phenotypic values; *T* is a matrix of fixed effects, including sex, farm of origin, batch containing the year-season effect, and the top three principal components with the corresponding effect, *w*_*i*_; *P*_*j*_ is the genotype matrix of *j* pseudo quantitative trait nucleotides (QTNs), which was used as the fixed effects; and *q*_*j*_ is the corresponding effect. *m*_*k*_ is a vector of genotypes for the *k*th marker to be tested, and *h*_*k*_ is the corresponding effect. *e* is the residual effect vector with distribution, $\text{e}∼N\left(0,I\sigma_{e}^{2}\right)$, where $\sigma_{e}^{2}$ represents the residual variance. The random effect model was used to select the most appropriate pseudo QTNs. The model can be written as follows:

y=u+e,

where *y* is the vector of the phenotypic values of meat quality; *u* is the genetic effects defined by $\text{u}∼N\left(0,2K\sigma_{u}^{2}\right)$, where *K* is the kinship matrix defined by pseudo QTNs, and $\sigma_{u}^{2}$ is an unknown genetic variance; and *e* is the residual effect vector.

The MLM can be written as follows:

y=Wb+Za+Sc+e,

where *y* is the vector of phenotypes of each D (LY) pig, *a* is the vector of the same fixed effects as those in the FarmCPU model, *b* is the vector of the SNP substitution effects, and *c* is the vector of random additive genetic effects with $\text{a}∼N\left(0,G\sigma_{a}^{2}\right)$, where *G* is the genomic relationship matrix, and $\sigma_{a}^{2}$ is the unknown additive variance. *W*, *Z*, and *S* are the incidence matrices for *b*, *a*, and *c*, respectively. Because the Bonferroni correction was too strict, the genome-wide significant thresholds were set as *p* < 1/N, where N was the number of SNPs tested in the analyses as per previous studies (Liu et al., 2015; Xiong et al., 2015; Ding et al., 2019). In this study, N was 34,057, and the significant threshold was set to 2.94E^–5^. Phenotypic correlations among the meat traits were calculated within the R statistical environment and used to determine whether they reflected the relationships between the GWAS results.

### Annotation of Candidate Genes

Potential candidate genes were identified within 500 kb upstream and downstream of the genome-wide significant SNPs on the Sus scrofa11.1 genome from the Ensembl database^2^. Candidate genes were then selected for traits according to their biological function.

### Haplotype Block Analysis

Haplotype block analysis was performed with Haploview software. Linkage disequilibrium blocks were defined using Haploview with the default parameters (Gabriel et al., 2002) based on SNPs with MAF values > 0.05, Mendelian errors < 2, and *p* in the HWE test < 10^–3^.

## Results

### Phenotype Description and Correlation Among Meat Traits

Tables 2, 3 summarizes the statistical information and genomic heritability of the meat-quality traits. Supplementary Figure 1 shows the trait distributions. The mean values for moisture, IMF, conductivity, pH, marbling score, and meat color were 71.1%, 2.43%, 2.65 mS, 6.36, 3.41, and 3.74, respectively. The genomic heritability estimates for moisture, IMF, conductivity, pH, marbling score and meat color were 0.48, 0.31, 0.13, 0.39, 0.37, and 0.55, respectively. Table 4 shows the phenotypic correlation coefficients for moisture, IMF, conductivity, pH, marbling score, and meat color. Significant positive correlations were found between pH and marbling score (*r* = 0.43; *p* < 0.01), meat color and moisture (*r* = 0.59; *p* < 0.01), and marbling score and IMF (*r* = 0.20; *p* < 0.01). Moisture was significantly negatively correlated with IMF (*r* = −0.41; *p* < 0.01), pH (*r* = −0.44; *p* < 0.01), and marbling score (*r* = −0.32; *p* < 0.01).

**TABLE 2:** Descriptive statistics for meat-quality traits of 582 pigs.

**Traits**	***N***	**Mean**	**SD**	**CV/%**	**Min.**	**Max.**
Moisture, %	519	71.10	2.10	2.95	63.57	75.53
IMF, %	522	2.43	0.87	35.68	0.06	5.20
Conductivity, mS	550	2.65	0.53	19.99	1.63	4.87
pH	578	6.36	0.37	5.74	5.29	6.99
Marbling (1–5)	582	3.41	0.61	17.82	2.00	5.00
Meat color (1–5)	581	3.74	0.55	14.71	1.50	5.25

*IMF, intramuscular fat; h_2_, heritability estimates.*

**TABLE 3:** Estimation of genetic parameters for meat quality.

**Traits**	**Additive genetic variance (SE)**	**Residual variance (SE)**	**h^2^ (SE)**
Moisture	1.17 (0.25)	1.29 (0.19)	0.48 (0.08)
IMF	0.21 (0.06)	0.47 (0.06)	0.31 (0.08)
Conductivity	0.04 (0.02)	0.25 (0.02)	0.13 (0.07)
pH	0.04 (0.01)	0.06 (0.01)	0.39 (0.08)
Marbling	0.12 (0.03)	0.20 (0.02)	0.37 (0.08)
Meat color	0.11 (0.02)	0.09 (0.01)	0.55 (0.08)

*SE, standard error.*

**TABLE 4:** Correlation coefficients of meat-quality trait phenotypes in the pigs.

**Traits**	**Moisture**	**IMF**	**Conductivity**	**pH**	**Marbling**	**Meat color**
Moisture						
IMF	−0.416**					
Conductivity	0.038	–0.041				
pH	−0.444**	0.078	−0.448**			
Marbling	−0.326**	0.203**	−0.212**	0.433**		
Meat color	0.595**	−0.137**	–0.050	−0.260**	−0.083*	

*p < 0.05; **p < 0.01. IMF, intramuscular fat.

### Significantly Associated SNPs Identified via GWAS and Functional Analysis

**FIGURE 1:**
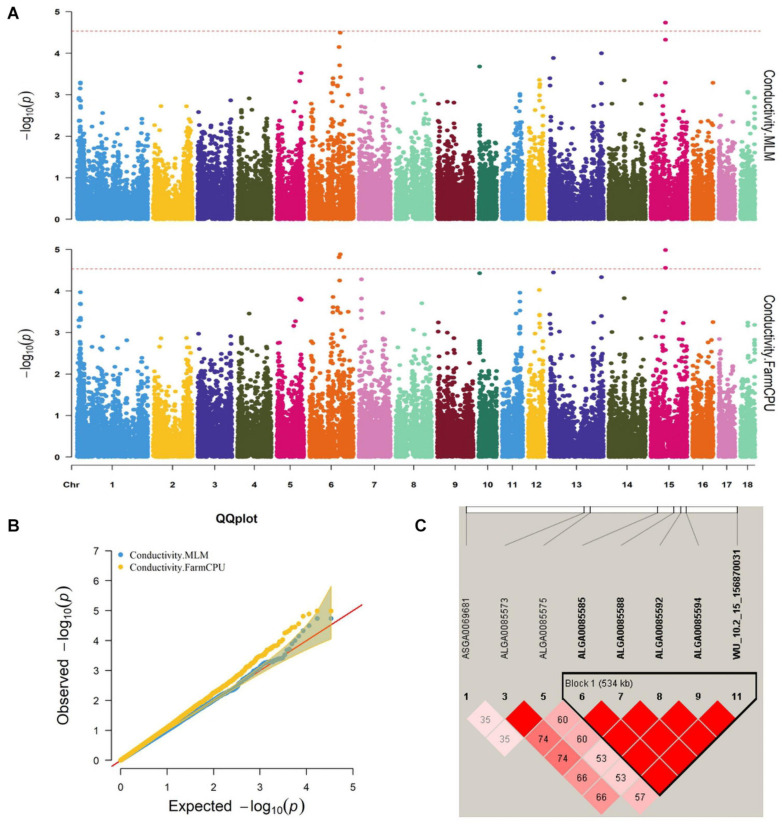
**(A)** Manhattan plots. **(B)** Quantile–quantile (QQ) plots of the mixed linear model (MLM) and fixed and random model Circulating Probability Unification (FarmCPU) analyzed for conductivity traits in D (LY) pigs. **(C)** Haplotype blocks on SSC15, including all significant conductivity-associated single-nucleotide polymorphisms (SNPs).

**FIGURE 2:**
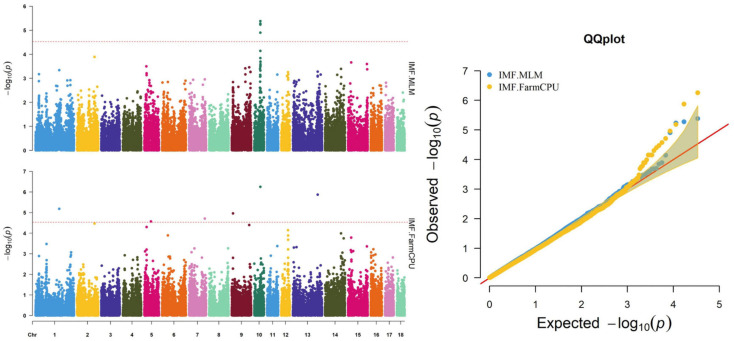
Manhattan and quantile–quantile (QQ) plots of the MLM and FarmCPU analyzed for IMF traits in D (LY) pigs.

**FIGURE 3:**
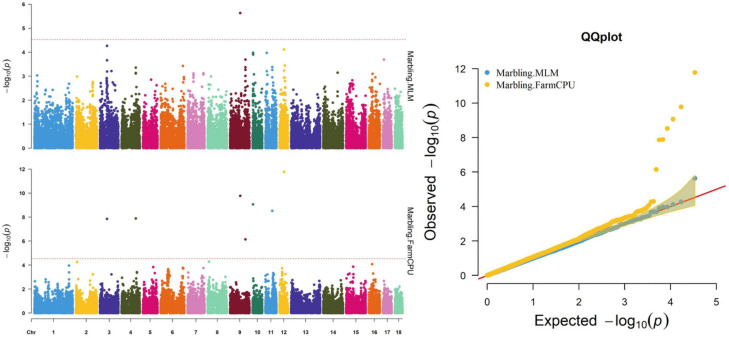
Manhattan and quantile–quantile (QQ) plots of the MLM and FarmCPU model analyzed for marbling score in D (LY) pigs.

**FIGURE 4:**
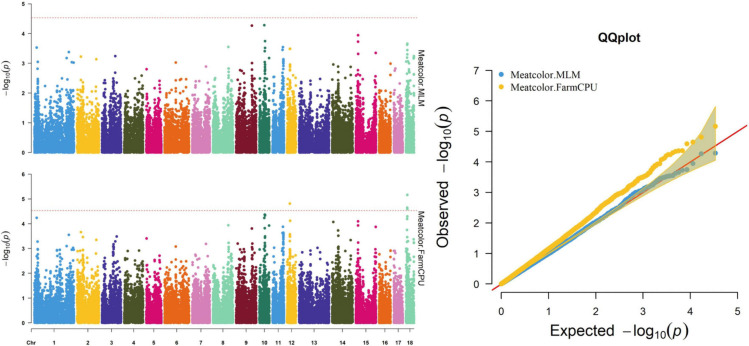
Manhattan and quantile–quantile (QQ) plots of the MLM and FarmCPU models analyzed for meat color in D (LY) pigs.

**FIGURE 5:**
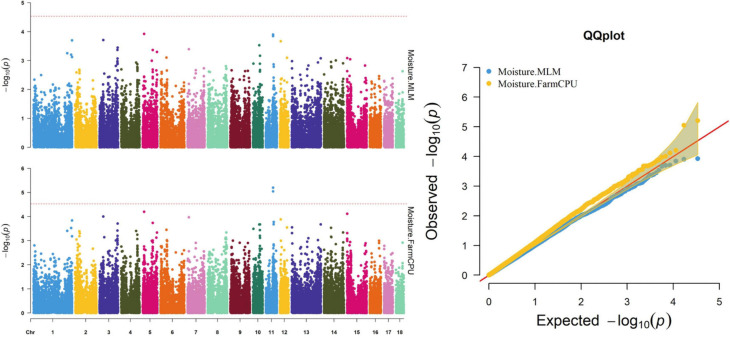
Manhattan and quantile–quantile (QQ) plots of the MLM and FarmCPU model analyzed for moisture in D (LY) pigs.

**FIGURE 6:**
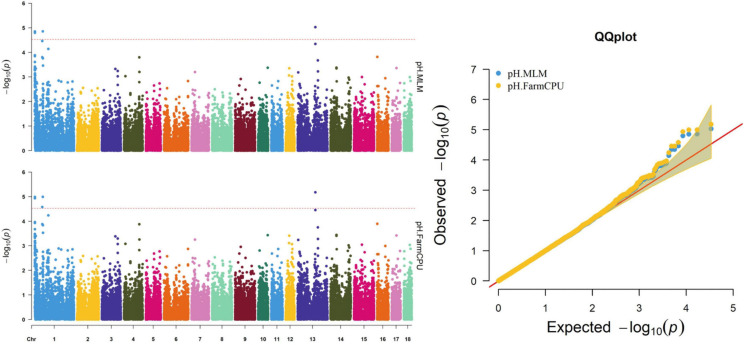
Manhattan and quantile–quantile (QQ) plots of the MLM and FarmCPU analyzed for pH in D (LY) pigs.

**FIGURE 7:**
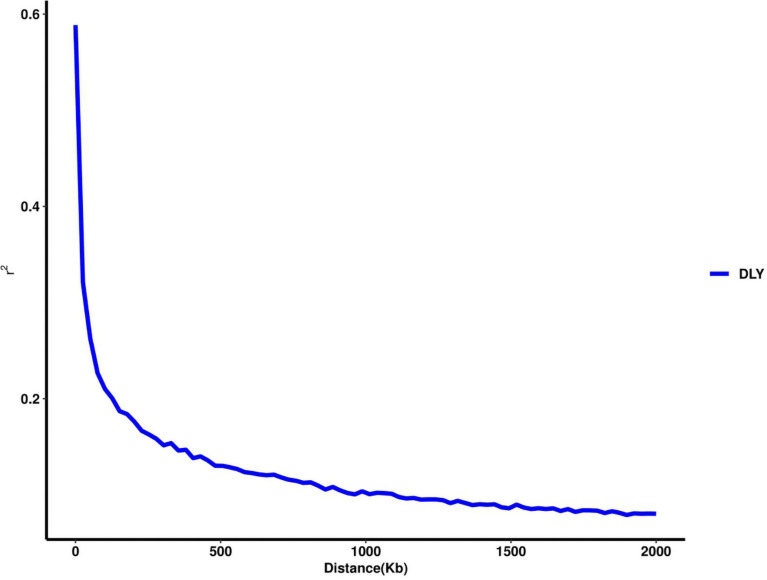
The linkage disequilibrium decay in populations of D (LY).

Thirty-two SNPs were identified as significant for the traits investigated (Figures 1–6). Among the detected SNPs, five, nine, seven, four, two, and five were associated with conductivity, IMF, marbling score, meat color, moisture, and pH, respectively. In addition, linkage disequilibrium (LD) analysis was performed by using the data of the D (LY) population, and the results are shown in Figure 7. The results show that LD decay tends to be stable statuses when the distance is 1 Mb. Thus, genes that located within 1 Mb near the significant SNPs are identified as potential candidate genes for traits. In this study, 140 functional genes located within 1 Mb of the significant SNPs were considered potential candidate genes (Supplementary Table 2). Eight genes were selected as candidate genes for meat-quality traits according to their biological functions.

### Conductivity

Five significant SNPs for conductivity were identified on chromosomes 6 and 15 (Figure 1A). Table 5 provides detailed information on the significant SNPs, including the SNP, chromosome (Chr), location (bp), *P*-value, whether the SNP is located on or flanking the gene, and distance between the flanking genes and significant SNPs. Three significant SNPs were located within a 0.20-Mb segment (from 56.34 to 56.54 Mb) on SSC15. Among them, the two most significant, ALGA0085588 and ALGA0085585, were located within *HERC2* and 93.3 kb upstream from *HERC2*, respectively, and were detected by both the MLM and FarmCPU. ALGA0085594 was also located within *HERC2* via the FarmCPU method. These three significant SNPs on SSC15 were in a 534-kb haplotype block (Figure 1C). The other significant SNPs, ASGA0083580 and DRGA0006706, were, respectively, located within *FHOD3* and 53.2 kb upstream from *DSG1* on SSC6.

**TABLE 5:** Genome-wide significant conductivity-associated SNPs.

**SNP**	**Chr^1^**	**Location (bp)**	**MAF**	***P* value**	**Located gene**	**Flanking genes**	**Distance^2^**	**Method^3^**
DRGA0006706	6	115,184,412	0.14	1.53E-05	–	*DSC1/DSG1*	−108,315/+53,265	I
ASGA0083580	6	120,435,160	0.06	1.30E-05	*FHOD3*	*MOCOS/TPGS2*	−417,040/+154,871	I
ALGA0085585	15	56,344,774	0.40	1.02E-05/1.82E-05	–	*MFHAS1/HERC2*	−49,683/+93,333	I, II
ALGA0085588	15	56,452,924	0.40	1.02E-05/1.82E-05	*HERC2*	*MFHAS1/ENSSSCG00 000047765*	−157,833/+361,732	I, II
ALGA0085594	15	56,538,806	0.33	2.75E-05	*HERC2*	*MFHAS1/ENSSSCG0 0000047765*	−243,715/+275,850	I

*^1^SNP located on the Sus scrofa Build 11.1 assembly.*

*^2^SNP designated as within a gene distance from the flanking gene-coding region in the Sus scrofa Build 11.1 assembly.*

*^3^Method numbers (I, II) represent the FarmCPU and MLM, respectively.*

### IMF

The MLM and FarmCPU methods identified nine significant SNPs associated with IMF (Figure 2 and Table 6). Among these significant SNPs, four (WU_10.2_10_48312614, WU_10.2_10_47748520, DRGA00 10501, and WU_10.2_10_48118152) were located within a 0.50-Mb segment (from 43.10 to 43.60 Mb) on SSC10. The most significant SNP (WU_10.2_10_48312614) was identified by both models and was located within an intron of *ST8SIA6*. ALGA0006955, ALGA0031885, H3GA0023123, DBWU0000868, and ASGA0059395 were located on SSC1, 5, 7, 9, and 13, respectively.

**TABLE 6:** Genome-wide significant SNPs associated with IMF.

**SNP**	**Chr^1^**	**Location (bp)**	**MAF**	***P* value**	**Located gene**	**Flanking genes**	**Distance^2^**	**Method^3^**
ALGA0006955	1	169,163,416	0.09	6.56E-06	–	*ENSSSCG0000004 5715/NR2E3*	−362,891/+197,313	I
ALGA0031885	5	47,014,709	0.24	2.66E-05	*ITPR2*	*INTS13/–*	−154,156/–	I
H3GA0023123	7	112,784,720	0.15	1.94E-05	–	*RPS6KA5 ENSSSCG0000 0002438*	−31,103/46,939	I
DBWU0000868	9	8,933,427	0.12	1.09E-06	*POLD3*	*LIPT2/CHRDL2*	−92,597/+80,680	I
WU_10.2_10_48312614	10	43,603,091	0.38	5.59E-07/4.15E-06	*ST8SIA6*	*VIM/ENSSSCG000 00046521*	−76,919/180,018	I, II
WU_10.2_10_47748520	10	43,105,103	0.37	1.25E-05	*CUBN*	*ENSSSCG0000004 8231/TRDMT1*	−20,418/329,341	II
DRGA0010501	10	43,457,312	0.20	5.82E-06	*TRDMT1*	*CUBN/VIM*	−11,444/+58,156	II
WU_10.2_10_48118152	10	43,496,534	0.15	5.30E-06	*TRDMT1*	*CUBN/VIM*	−81,921/+20,773	II
ASGA0059395	13	177,464,038	0.43	1.33E-06	*ROBO2*	*ENSSSCG00000046597/–*	−462,956/–	I

*^1^SNP located on the Sus scrofa Build 11.1 assembly.*

*^2^SNP designated as within a gene distance from the flanking gene-coding region in the Sus scrofa Build 11.1 assembly.*

*^3^Method numbers (I, II) represent the FarmCPU and MLM, respectively.*

### Marbling

Both models identified seven significant SNPs associated with marbling (Figure 3 and Table 7). One SNP (M1GA0013120) was identified by only MLM. The most significant SNP (WU_10.2_12_33077453) was located 141.0 kb upstream of *ANKFN1*.

**TABLE 7:** Genome-wide significant SNPs associated with marbling score.

**SNP**	**Chr^1^**	**Location (bp)**	**MAF**	***P* value**	**Located gene**	**Flanking genes**	**Distance^2^**	**Method^3^**
ALGA0018939	3	50,684,383	0.11	1.38E-08	–	–	–	I
WU_10.2_4_111643880	4	101,653,530	0.36	1.29E-08	*HAO2*	*ENSSSCG00000 006719/WARS2*	−79,702/+133970	I
M1GA0013120	9	72,761,757	0.12	1.65E-10/2.32E-06	*–*	*ENSSSCG0000004 8637/SAMD9*	−222,088/+202058	I, II
ASGA0044293	9	110,280,507	0.49	7.12E-07	–	*ENSSSCG00000034739/ENSSSCG00000015460*	−470,585/+179,469	I
WU_10.2_10_5204072	10	3,387,068	0.46	8.68E-10	–	*BRINP3/–*	−19,445/–	I
WU_10.2_11_53938211	11	49,300,307	0.26	2.99E-09	*MYCBP2*	*FBXL3/SCEL*	−149,298/+351,366	I
WU_10.2_12_33077453	12	32,245,751	0.43	1.67E-12	–	*ENSSSCG00000025681/ANKFN1*	−89,735/+141,018	I

*^1^SNP located on the Sus scrofa Build 11.1 assembly.*

*^2^SNP designated as within a gene distance from the flanking gene-coding region in the Sus scrofa Build 11.1 assembly.*

*^3^Method numbers (I, II) represent the FarmCPU and MLM, respectively.*

### Meat Color

The FarmCPU identified four significant SNPs associated with meat color (Table 8 and Figure 4); the MLM identified no SNPs for meat color. Three of the four significant SNPs were located within 0.39 Mb (from 9.20 to 9.59 Mb) on SSC18. The most significant SNPs, M1GA0023045 and WU_10.2_18_10095600, were located 22.2 kb from *KDM7A* on SSC18. The other significant SNPs (WU_10.2_12_18572268, ASGA0078801, and WU_10.2_18_10095600) were located within an intron of *NMT1* on SSC12 and *DENND2A* and *KDM7A* on SSC18, respectively.

**TABLE 8:** Genome-wide significant SNPs associated with meat color.

**SNP**	**Chr^1^**	**Location (bp)**	**MAF**	***P* value**	**Located gene**	**Flanking genes**	**Distance^2^**	**Method^3^**
WU_10.2_12_18572268	12	18,323,553	0.43	1.53341E-05	*NMT1*	*PLCD3/C1QL1*	−21,069/+86,557	I
ASGA0078801	18	9,196,074	0.19	2.23566E-05	*DENND2A*	*ADCK2/MKRN1*	−23,041/+137,610	I
M1GA0023045	18	9,559,135	0.44	6.84623E-06	*–*	*SLC37A3/KDM7A*	−106,542/+22,249	I
WU_10.2_18_10095600	18	9,589,537	0.47	2.53367E-05	*KDM7A*	*SLC37A3/PARP12*	−136,944/+85,407	I

*^1^SNP located on the Sus scrofa Build 11.1 assembly.*

*^2^SNP designated as within a gene distance from the flanking gene-coding region in the Sus scrofa Build 11.1 assembly.*

*^3^Method numbers (I, II) represent the FarmCPU and MLM, respectively.*

### Moisture

The FarmCPU identified two significant SNPs associated with moisture; the MLM identified no significant SNPs associated with moisture (Table 9). Figure 5 shows the Manhattan and QQ plots. The significant SNPs, WU_10.2_11_56636318 and ALGA0062389, were, respectively, located 275.5 and 325.9 kb downstream from *NDFIP2*.

**TABLE 9:** Genome-wide significant SNPs associated with moisture.

**SNP**	**Chr^1^**	**Location (bp)**	**MAF**	***P* value**	**Located gene**	**Flanking genes**	**Distance^2^**	**Method^3^**
WU_10.2_11_56636318	11	51,835,854	0.42	6.26E-06	–	*NDFIP2/ENSSSCG00000051397*	−275,549/+307,411	I
ALGA0062389	11	51,886,282	0.26	8.98E-06	–	*NDFIP2/ENSSSCG00000051397*	−325,977/+256,983	I

*^1^SNP located on the Sus scrofa Build 11.1 assembly.*

*^2^SNP designated as within a gene distance from the flanking gene-coding region in the Sus scrofa Build 11.1 assembly.*

*^3^Method numbers (I, II) represent the FarmCPU and MLM, respectively.*

### pH

Five significant SNPs on SSC1 and SSC13 were significantly associated with pH (Table 10 and Figure 6). Among these SNPs, four (WU_10.2_1_934682, WU_10.2_1_974053, INRA0002536, and ASGA0099314) were identified via both the FarmCPU and MLM. The most significant SNP (ASGA0099314) was located within *ETV5*, a protein-coding gene.

**TABLE 10:** Genome-wide significant SNPs associated with pH.

**SNP**	**Chr^1^**	**Location (bp)**	**MAF**	***P* value**	**Located gene**	**Flanking genes**	**Distance^2^**	**Method^3^**
WU_10.2_1_934682	1	557,299	0.34	1.02E-05/1.40E-05	*PHF10*	*TCTE3/C6orf120*	−5,336/+18,419	I, II
WU_10.2_1_974053	1	596,709	0.35	1.16E-05/1.58E-05	*ENSSSCG00 000004008*	*C6orf120/THBS2*	−18,278/+258,321	I, II
ALGA0003423	1	52,262,327	0.45	2.63E-05	*–*	*RIMS1/KCNQ5*	−17,393/+173,107	I
INRA0002536	1	56,511,890	0.33	1.01E-05/1.39E-05	*–*	*ENSSSCG00000050040/ENSSSCG00000029003*	−44,660/+17,7178	I, II
ASGA0099314	13	123,889,649	0.37	6.60E-06/9.35E-06	*ETV5*	*ENSSSCG0000003 9758/DGKG*	−74,040/72,114	I, II

*^1^SNP located on the Sus scrofa Build 11.1 assembly.*

*^2^SNP designated as within a gene distance from the flanking gene-coding region in the Sus scrofa Build 11.1 assembly.*

*^3^Method numbers (I, II) represent the FarmCPU and MLM, respectively.*

## Discussion

As living standards continuously improve, consumers have higher expectations and more rigorous demands regarding meat quality. Consequently, meat quality is becoming an important trait in the swine industry and a major issue for pig breeding programs (Moeller et al., 2010; Gallardo et al., 2012; Nonneman et al., 2013). With the development of SNP arrays, GWAS analyses have become important for improving meat quality in the swine industry. For example, a previous study showed that several candidate genes, including *MC4R*, *IGF2*, *ADRB3*, and *ATP1A2*, heavily affected meat quality (Lu et al., 2018; Mármol Sánchez et al., 2020). Researchers showed that the AG genotype of *ADRB3* had a higher marbling score and that it could be an important marker for improving marbling scores (Kenchaiwong et al., 2020). In this study, we performed a GWAS of meat-quality traits on crossbred commercial D (LY) pigs and detected candidate genes and markers to improve meat-quality traits.

In this work, the genomic heritability of the meat-quality traits ranged from 0.13 to 0.55, which was similar to the results of a previous study (Miar et al., 2014). The estimated heritabilities of these meat-quality traits were of low or moderate magnitude, showing that meat quality can be genetically improved. We identified 32 SNPs that were significantly associated with meat-quality traits in crossbred D (LY) pigs. Limited SNPs were analyzed, possibly owing to the sample size and hybrid nature of the three-way crossbred population. Previous studies identified nine SNPs for meat-quality traits in a population of 610 D (LY) pigs, and 28 SNPs were identified in a population of 336 purebred Chinese Erhualian pigs (Liu et al., 2015). Thus, the GWAS results may have been related to both the variety and population size of the pigs.

Notably, in addition to duplicating the QTL for meat quality found in a previous GWAS, we identified four novel QTLs. These four novel QTLs were located on a 0.20-Mb region (56.34–56.54 Mb) significantly associated with conductivity on SSC15, a 0.39-Mb region (9.19–9.58 Mb) significantly associated with meat color on SSC18, a 0.04-Mb region (0.56–0.60 Mb) significantly associated with pH on SSC1, and a 4.25-Mb region (52.26–56.51 Mb) on SSC1. Additionally, a 2.59-Mb region (51.89–49.30 Mb) on SSC11 was identified as being significantly associated with marbling and moisture, containing the significant SNPs WU_10.2_11_53938211 at 49.30 Mb for marbling, and WU_10.2_11_56636318 at 51.89 Mb and ALGA0062389 at 51.83 Mb for moisture. The results showed that some chromosomal regions might have diverse effects on meat-quality traits. Moreover, similar to the results of Luo et al. (2012), moderate correlation coefficients were identified between marbling and moisture (*r* = −0.33; *p* < 0.01). Thus, the correlation between traits might explain the pleiotropic effects in some regions.

We identified five significant SNPs as being significantly associated with conductivity. Two of these (ALGA0085585 and ALGA0085588) were identified by the FarmCPU and MLM and were located near *HECT and RLD domain-containing E3 ubiquitin protein ligase 2* (*HERC2*). ALGA0085594 was also located within *HERC2*. Previous research found that *ATP1A2* was strongly associated with muscle electrical conductivity because it encoded a subunit of the Na^+^/K^+^-ATPase responsible for maintaining an electrochemical gradient across the plasma membrane (Mármol Sánchez et al., 2020). Furthermore, *ATP1A2* polymorphisms were associated with fat-cut percentage (Fontanesi et al., 2012). The function of *HERC2* has been related to decreased body fat mass in mice. We speculated that *HERC2* likely affects the electrical conductivity by affecting fat metabolism in pigs. *Desmoglein 3* (*DSG3*), another candidate gene for conductivity, was located 0.1 Mb from the significant SNP, DRGA0006706, a protein-coding gene whose gene ontology annotations indicate that it is related to cytosolic metabolic processes (Drag et al., 2019) and calcium ion binding (Gaudet et al., 2011).

The ASGA0059395 SNP was located within *roundabout guidance receptor 2* (*ROBO2*) of the *ROBO* family. Some researchers showed that *ROBO2* was involved in fat metabolism, especially in fatty acid composition and includes C18:3_*IMF*_ (Sato et al., 2017). Furthermore, SNP WU_10.2_10_48312614 was detected via two methods and located 0.45 Mb upstream from *transmembrane protein 236* (*TMEM236*). No research has found *TMEM236* to be involved in fat metabolism, but its related genes, *transmembrane protein 120A* (*TMEM120A*) and *transmembrane protein 120B* (*TMEM120B*), affect adipocyte differentiation and metabolism in mice and are highly expressed in fat (Batrakou et al., 2015). Additionally, *transmembrane protein 60* (*TMEM60*) is another homologous gene associated with marbling fat in cattle (Lim et al., 2014). *TMEM236* is reportedly associated with fat color (Xia et al., 2016). Thus, *ROBO2* and *TMEM236* are strong potential candidate genes for IMF. Several researchers have reported a positive correlation between marbling and IMF (Luo et al., 2012; Ma et al., 2013), which is consistent with the results of this study. Similarly, fat metabolism also affects marbling. *Ankyrin repeat and fibronectin type III domain-containing 1* (*ANKFN1*), located 0.14 Mb from SNP WU_10.2_12_33077453, is involved in regulating fat androstenone levels (Drag et al., 2019) and might be an important potential candidate gene for marbling.

Meat color is a complex trait and is affected by pigment concentration, structural conditions of the muscle tissue, and the muscle acidification rate (Fan et al., 2008; Mármol Sánchez et al., 2020). In the present study, the SNPs M1GA0023045 and WU_10.2_18_10095600 on SSC18 located 106.5 and 139.6 kb upstream of *solute carrier family 37 member 3* (*SLC37A3*), respectively, were first associated with meat color. The related genes, *solute carrier family 15 member 4* (*SLC15A4*) and *solute carrier family 25 member 17* (*SLC25A17*), participate in regulating pork quality. Researchers reported that the *SLC15A4* c.658AA genotype had better water-holding capacity and reduced color b^∗^ and color L^∗^ (D’Astous-Pagé et al., 2017). *SLC25A17* was also associated with meat color in a previous study (Ma et al., 2013). Therefore, *SLC37A3* may be a potential candidate gene for meat color, although no reports have demonstrated its role in meat quality.

Meat moisture content was strongly negatively correlated with IMF content in our study, which was consistent with previous studies (Chin et al., 2012; Luo et al., 2012). Leaner meats generally contain more water because water is essential for protein synthesis and muscle building. In this study, the QTLs (from 51.84 to 51.89 Mb) on SSC11, including WU_10.2_11_56636318 and ALGA0062389, were identified via the FarmCPU model. Previous researchers found that the QTL on SSC11 was associated with IMF content, drip loss, and meat color score (Kim et al., 2005; Won et al., 2018). In this study, we, for the first time, identified the QTLs on SSC11 as being associated with moisture.

pH is an important meat-quality trait, is affected by glycogen metabolism, and can affect Pale-Soft-Exudative (PSE) and Dark-Firm-Dry (DFD) production. Studies have suggested that *PPP1R3B* is a candidate gene for pH because it affects glycogen by stimulating glycogen accumulation (Worby et al., 2008) and decreases muscle glycogen phosphorylase phosphatase activity (Doherty et al., 1995). *Regulating synaptic membrane exocytosis 1* (*RIMS1*), which plays a role in regulating voltage-gated calcium channels during neurotransmitter and insulin release in humans, was located 17.4 kb of ALGA0003423 on SSC1. This gene might regulate glycogen metabolism through insulin, thus affecting pork pH values. Furthermore, *insulin-like growth factor 2 mRNA-binding protein 2* (*IGF2BP2*), another candidate gene for pH, encodes a protein that binds the 5′-untranslated region of IGF2 mRNA and regulates its translation. It plays an important role in glycogen metabolism, and variation of this gene has been associated with susceptibility to diabetes (Cho et al., 2008). Thus, *RIMS1* and *IGF2BP2* may be potential candidate genes for pH based on their biological functions.

**FIGURE 8:**
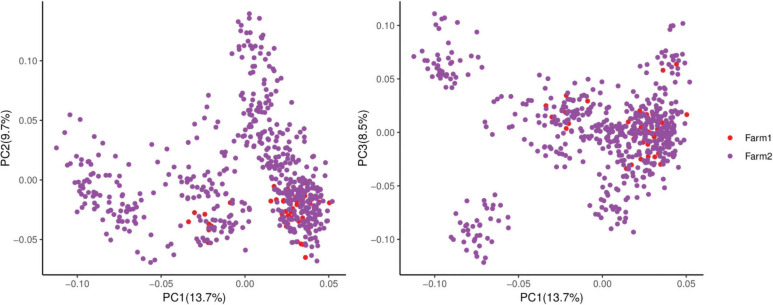
Population principal component analysis.

Many factors affect the validity of GWAS results. Population stratification is an important factor that can lead to false positives (Pearson and Manolio, 2008). Many studies have reported that adding group structure to GWAS models improved the accuracy of the results (Yu et al., 2006; Zhang et al., 2010). In this study, we performed a principal component analysis and obtained the eigenvalue decomposition of the genomic relationship matrix. The results of the principal component analysis are shown in Figure 8. The results showed that the first, second, and third principal components comprised 13.7, 9.7, and 8.5% of the total genomic variance, respectively. To eliminate the influence of population stratification, the top three principal component effects controlling the population genetic background were added into this research model. The number of statistical models was used to control false positives by adding population structure and the MLM that was most commonly used for GWAS. However, although the MLM reduced the incidence of false positives, it induced false negatives by over-fitting the model to a degree that enabled missing potentially important associations (Kaler et al., 2017). As shown in this study, although Manhattan plots from both MLM and Manhattan plots were similar in meat color and moisture, MLM leads to false-negative results, while FarmCPU can overcome the shortcomings of MLM and successfully identified SNPs or candidate gene for traits. Additionally, we used two models, the FarmCPU and MLM, to perform a GWAS for six meat-quality traits in 582 D (LY) pigs. Figures 1–6 show the QQ plots for meat traits in the different models. In the FarmCPU, the deflation factors for IMF, moisture, marbling, conductivity, meat color, and pH were 0.9, 1.1, 1.0, 1.1, 1.1, and 0.9, respectively; in the MLM, these factors were 1.0, 0.9, 1.0, 1.0, 0.9, and 0.9, respectively. We found no obvious population stratifications, and the populations could be managed well using the FarmCPU and MLM. Additionally, although we identified candidate genes for meat-quality traits from their biological function and proximity to significant SNPs (within 1 Mb), candidate genes may exist outside this distance. FarmCPU identified 29 of 32 significant SNPs. Moisture and meat color were not identified in the MLM, thus limiting its use in the present study. The FarmCPU found all candidate genes for meat-quality traits in this study, whereas the MLM only found half of these candidate genes. Previous studies also indicated that FarmCPU identified more candidate genes in both animals and plants because it better controlled for false negatives and false positives (Meng et al., 2017; Wang et al., 2018; Kaler et al., 2019; Bollinedi et al., 2020). Overall, the results suggested that the FarmCPU model worked well in detecting candidate genes, particularly for complex meat-quality traits.

## Conclusion

We conducted a GWAS for meat-quality traits in 582 D (LY) pigs using both FarmCPU and MLM. Thirty-two significant SNPs and several subsequent candidate genes were identified as being associated with meat-quality traits. The biological functions of the candidate genes aligned well with regulating the corresponding meat-quality traits. Furthermore, the FarmCPU worked well in identifying candidate genes, particularly for complex meat-quality traits. Overall, the significant SNPs and candidate genes identified herein may benefit pig-breeding programs and contribute to further improving meat quality.

## Data Availability Statement

In present study, the SNP genotype data was deposited in the Figshare Repository (https://figshare.com/s/7c316a6828f238 af0f12).

## Ethics Statement

The animal study was reviewed and approved by the Institutional Animal Care and Use Committee of Foshan University. Written informed consent was obtained from the owners for the participation of their animals in this study.

## Author Contributions

YZ developed the experimental design. GG was in charge of the experiment execution and data collection and wrote the manuscript. GG and NG processed the data and performed the statistical analysis. All authors made the final revisions, were involved in developing, writing, and commenting on the manuscript, and read and approved the final manuscript.

## Conflict of Interest

NG, SL, LZ, WJ, and YZ were employed by the company Guangxi Yangxiang Co., Ltd. The remaining authors declare that the research was conducted in the absence of any commercial or financial relationships that could be construed as a potential conflict of interest.

## Footnotes

1 https://www.animalgenome.org/cgi-bin/QTLdb/SS/summary

2 http://asia.ensembl.org/
