# Supplementary material for: Genome-Wide Association Study of Meat Quality Traits in a Three-Way Crossbred Commercial Pig Population
Source: Front Genet. 2021 Mar 17;12:614087. doi: 10.3389/fgene.2021.614087 (PMC8010252; doi:10.3389/fgene.2021.614087)
Supplement: Supplementary file 1 [file Table_1.DOCX]

**TABLE S1 |** Distributions of SNPs after quality control and the average distance between adjacent SNPs on each chromosome.

| **Chromosome** | **SNP number** | **Pysical map(Mb)^1^** | **Mb/SNP** |
| --- | --- | --- | --- |
| 1 | 3082 | 274.33 | 0.0890 |
| 2 | 2378 | 151.94 | 0.0639 |
| 3 | 2168 | 132.85 | 0.0613 |
| 4 | 2206 | 130.91 | 0.0593 |
| 5 | 1660 | 104.53 | 0.0630 |
| 6 | 2136 | 170.84 | 0.0800 |
| 7 | 2126 | 121.84 | 0.0537 |
| 8 | 2176 | 138.97 | 0.0639 |
| 9 | 2329 | 139.51 | 0.0599 |
| 10 | 1093 | 69.36 | 0.0635 |
| 11 | 1332 | 79.17 | 0.0594 |
| 12 | 994 | 61.60 | 0.0620 |
| 13 | 2663 | 208.33 | 0.0782 |
| 14 | 2348 | 141.76 | 0.0604 |
| 15 | 2057 | 140.41 | 0.0683 |
| 16 | 1308 | 79.94 | 0.0611 |
| 17 | 1054 | 63.49 | 0.0602 |
| 18 | 947 | 55.98 | 0.0591 |

SNP single nucleotide polymorphisms

^1^The physical size is based on Sus scrofa Build 11.1
