# Supplementary material for: Genome-Wide Association Study of Meat Quality Traits in a Three-Way Crossbred Commercial Pig Population
Source: Front Genet. 2021 Mar 17;12:614087. doi: 10.3389/fgene.2021.614087 (PMC8010252; doi:10.3389/fgene.2021.614087)
Supplement: Supplementary file 2 [file Table_2.docx]

**Table S2: Annotated genes with less than 1 Mb of significant SNPs**

| **Traits** | **SNP** | **Chr** | **Position** | **Adjacent genes (±1 Mb)** |
| --- | --- | --- | --- | --- |
| Conductivity | DRGA0006706 | 6 | 115184412 | DSC2, DSC1, DSG1, DSG4, DSG3, DSG2, TTR, B4GALT6 |
|  | ASGA0083580 | 6 | 120435160 | MOCOS,FHOD3,TPGS2, ENSSSCG00000042283，KIAA1328 |
|  | ALGA0085585 | 15 | 56344774 | PPP1R3B, ERI1, MFHAS1, HERC2, ENSSSCG00000047765 |
|  | ALGA0085588 | 15 | 56452924 | PPP1R3B, ERI1, MFHAS1, HERC2, ENSSSCG00000047765, ENSSSCG00000016393 |
|  | ALGA0085594 | 15 | 56538806 | ERI1, MFHAS1, HERC2, ENSSSCG00000047765, ENSSSCG00000016393, ENSSSCG00000016392, AMER3 |
| IMF | ALGA0006955 | 1 | 169163416 | LRRC49, THSD4, ENSSSCG00000045715, NR2E3, ENSSSCG00000037781, MYO9A, SENP8, ENSSSCG00000026486, ENSSSCG00000024102 |
|  | ALGA0031885 | 5 | 47014709 | [STK38L](http://asia.ensembl.org/Sus_scrofa/Gene/Summary?db=core;g=ENSSSCG00000022288;r=5:46490410-46632573;v=rs81384035;vdb=variation;vf=333785), ENSSSCG00000042702, MED21, TM7SF3, ENSSSCG00000036398, FGFR1OP2, INTS13, ITPR2 |
|  | H3GA0023123 | 7 | 112784720 | TTC7B, RPS6KA5, ENSSSCG00000002438, GPR68, CCDC88C, PPP4R3A, CATSPERB, ENSSSCG00000048573 |
|  | DBWU0000868 | 9 | 8933427 | C2CD3,PPME1,P4HA3,PGM2L1,KCNE3,LIPT2,POLD3,CHRDL2,RNF169,XRRA1,  SPCS2,NEU3 |
|  | WU_10.2_10_48312614 | 10 | 43603091 | CUBN, TRDMT1, VIM, ST8SIA6, ENSSSCG00000046521, HACD1, STAM, TMEM236 |
|  | WU_10.2_10_47748520 | 10 | 43105103 | KIF5B, ENSSSCG00000051254, EPC1, ENSSSCG00000022045, ENSSSCG00000048231, CUBN, TRDMT1, VIM, ST8SIA6 |
|  | DRGA0010501 | 10 | 43457312 | ENSSSCG00000022045, ENSSSCG00000048231, CUBN, TRDMT1, VIM, ST8SIA6, ENSSSCG00000046521, HACD1, STAM |
|  | WU_10.2_10_48118152 | 10 | 43496534 | ENSSSCG00000022045, ENSSSCG00000048231, CUBN, TRDMT1, VIM, ST8SIA6, ENSSSCG00000046521, HACD1, STAM |
|  | ASGA0059395 | 13 | 177464038 | ENSSSCG00000046597, ROBO2 |
| Marbling | ALGA0018939 | 3 | 50684383 | -- |
|  | WU_10.2_4_111643880 | 4 | 101653530 | ENSSSCG00000040490, ENSSSCG00000045266, ENSSSCG00000044715, ADAM30, REG4, HMGCS2, PHGDH, ZNF697, ENSSSCG00000006719, HAO2, WARS2, ENSSSCG00000006725 |
|  | M1GA0013120 | 9 | 72761757 | ANKIB1, ENSSSCG00000027356, GATAD1, ENSSSCG00000051578, PEX1, RBM48, ENSSSCG00000015316, FAM133B, CDK6, ENSSSCG00000048637, SAMD9, HEPACAM2, VPS50 |
|  | ASGA0044293 | 9 | 110280507 | ENSSSCG00000034739/ENSSSCG00000015460 |
|  | WU_10.2_10_5204072 | 10 | 3387068 | BRINP3 |
|  | WU_10.2_11_53938211 | 11 | 49300307 | KCTD12,ACOD1,ENSSSCG00000031288,FBXL3,MYCBP2,SCEL |
|  | WU_10.2_12_33077453 | 12 | 32245751 | TMEM100, ENSSSCG00000017608, ENSSSCG00000025681, ANKFN1 |
| Meat color | WU_10.2_12_18572268 | 12 | 18323553 | LYZL6, ENSSSCG00000017577, ENSSSCG00000041162, ENSSSCG00000051222, PLEKHM1, MAP3K14, ENSSSCG00000017331, ENSSSCG00000017332, FMNL1, DCAKD, HEXIM2, HEXIM1, ACBD4, PLCD3, NMT1, C1QL1, KIF18B, GFAP, FAM187A, ENSSSCG00000017345, EFTUD2, HIGD1B, GJC1, ADAM11, DBF4B, CCDC43, MEIOC, FZD2, ITGA2B |
|  | ASGA0078801 | 18 | 9196074 | TMEM178B, MRPS33, ENSSSCG00000041343, BRAF, NDUFB2, ADCK2, DENND2A, MKRN1, RAB19, SLC37A3, KDM7A, PARP12 |
|  | M1GA0023045 | 18 | 9559135 | BRAF, NDUFB2, ADCK2, DENND2A, MKRN1, RAB19, SLC37A3, KDM7A, PARP12, TBXAS1, HIPK2 |
|  | WU_10.2_18_10095600 | 18 | 9589537 | BRAF, NDUFB2, ADCK2, DENND2A, MKRN1, RAB19, SLC37A3, KDM7A, PARP12, TBXAS1, HIPK2 |
| Moisture | WU_10.2_11_56636318 | 11 | 51835854 | RBM26, NDFIP2, ENSSSCG00000051397, SPRY2, ENSSSCG00000050522 |
|  | ALGA0062389 | 11 | 51886282 | RBM26, NDFIP2, ENSSSCG00000051397, SPRY2, ENSSSCG00000050522 |
| pH | WU_10.2_1_934682 | 1 | 557299 | FAM120B, DLL1, ENSSSCG00000047845, ERMARD, TCTE3, PHF10, C6orf120, ENSSSCG00000004008, THBS2 |
|  | WU_10.2_1_974053 | 1 | 596709 | FAM120B, DLL1, ENSSSCG00000047845, ERMARD, TCTE3, PHF10, C6orf120, ENSSSCG00000004008, THBS2 |
|  | ALGA0003423 | 1 | 52262327 | RIMS1, KCNQ5 |
|  | INRA0002536 | 1 | 56511890 | ORC3, ENSSSCG00000004307, SPACA1, CNR1, ENSSSCG00000050040, ENSSSCG00000029003, RNGTT |
|  | ASGA0099314 | 13 | 123889649 | LIPH, SENP2, ENSSSCG00000051486, IGF2BP2, TRA2B, ENSSSCG00000039758, ETV5, DGKG, ENSSSCG00000047010, CRYGS, TBCCD1 |
