# Supplementary material for: Genome-Wide Association Study of Meat Quality Traits in a Three-Way Crossbred Commercial Pig Population
Source: Front Genet. 2021 Mar 17;12:614087. doi: 10.3389/fgene.2021.614087 (PMC8010252; doi:10.3389/fgene.2021.614087)
Supplement: Supplementary file 3 [file Data_Sheet_1.PDF]

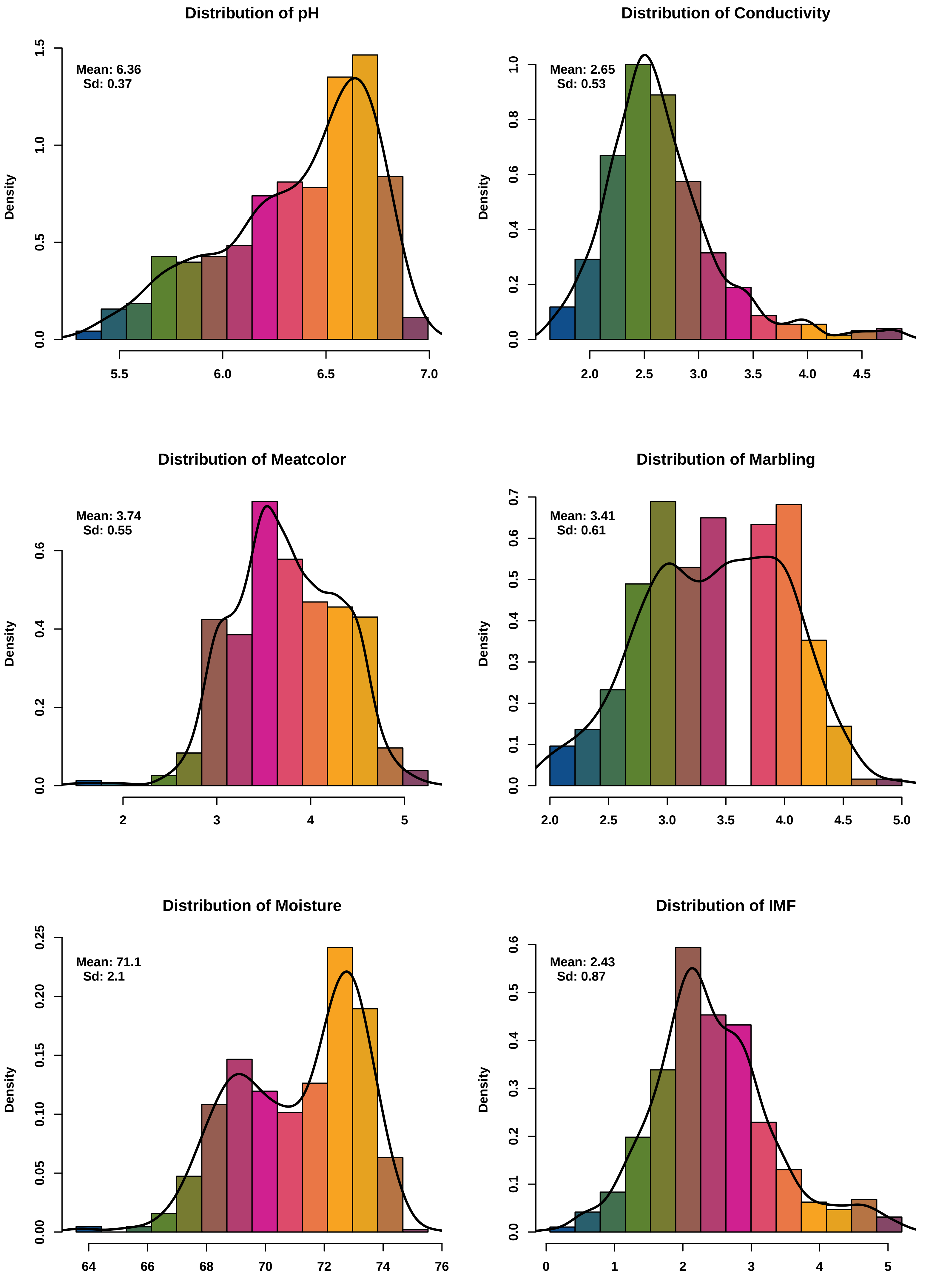
Distribution of pH
Mean: 6.36
Sd: 0.37
Density
Distribution of Conductivity
Mean: 2.65
Sd: 0.53
Density
Distribution of Meatcolor
Mean: 3.74
Sd: 0.55
Density
Distribution of Marbling
Mean: 3.41
Sd: 0.61
Density
Distribution of Moisture
Mean: 71.1
Sd: 2.1
Density
Distribution of IMF
Mean: 2.43
Sd: 0.87
Density
